# Parental age at conception on mouse lemur’s offspring longevity: Sex-specific maternal effects

**DOI:** 10.1371/journal.pone.0265783

**Published:** 2022-12-29

**Authors:** Perret Martine, Anzeraey Aude

**Affiliations:** UMR 7179, Adaptive mechanisms and Evolution, MECADEV, Brunoy, France; University of Massachusetts Medical School, UNITED STATES

## Abstract

Parental age at conception often influences offspring’s longevity, a phenomenon referred as the “Lansing effect” described in large variety of organisms. But, the majority of the results refer to the survival of juveniles, mainly explained by an inadequate parental care by the elderly parents, mostly the mothers. Studies on the effect of parental age on offspring’s longevity in adulthood remain few, except in humans for whom effects of parental age vary according to statistical models or socioeconomic environments. In a small primate in which the longevity reaches up to 13 years, we investigated the effects of parental age at conception on the longevity of offspring (N = 278) issued from parents with known longevity. None of the postnatal parameters (body mass at 30 and 60 days after birth, size and composition of the litter) influenced offspring’s longevity. Mothers’ age at conception negatively affected offspring’s longevity in males but not in females. By contrast, fathers’ age at conception did not influence offspring’s longevity. Finally, the longevity of female offspring was significantly positively related to the longevity of both parents. Compared with current studies, the surprisingly minor effect of fathers ‘age was related to the high seasonal reproduction and the particular telomere biology of mouse lemurs.

## Introduction

Longevity varies greatly among individuals and increasing evidence suggests that parental age affects longevity of offspring: the longevity of offspring of older parents is shorter than that of the offspring of younger parents. This phenomenom referred as the “Lansing effect” has been decribed in many taxa including more than 300 species [1, 2]. The underlying mechanisms mainly rely on the age-related changes of germ cells and particularly of telomeres [3, 4]. However, the majority of results confirming the “Lansing effect” refer to juveniles’ survival, which is mainly explained by an inadequate parental care of aged parents. Depending on the father’s role in parental care, sex differences exist for the effects of mothers ‘age on juveniles’ survival. In birds, juveniles’ survival may be favored by older females, but it also largely depends on the resources provided by the father [5–9]. In fact, the influence of parent’s age can be reduced, even absent, when the environmental conditions are favorable. In mammals, most of studies refer to the negative effects of mothers’ age on the pre-adult survival [2], whereas a few studies reported the role of the paternal age at conception on offspring survival [10, 11].

In humans, numerous recent studies have tested the “Lansing effect” on pre-industrial populations [10, 11] or on modern societies [12–14]. Despite extremely large samples (from 10.000 to more than five millions of people), contradictory results on the effects of parental age on the longevity of children were described, either negative or positive, depending on the statistical models, on the methods used or on the socio-economic environment to which the lengthening human longevity plays a preponderant role [15, 16].

Among non-human primates, studies on the effects of parental age on adult survival remain rare because of their great longevity and their low reproduction rate. By contrast, Malagasy prosimians constitute a more suitable model owing to their higher reproduction rate and their lower longevity. Among Malagasy species, juvenile survival until weaning is maximal when mothers are middle-aged but rapidly decreases reaching less than 20% for oldest females [17–19]. However, few studies investigated the relationship between parent’s age and longevity of offspring at adulthood.

To test the “Lansing effect”, we focused on the grey mouse lemur, a Malagasy primate. In captivity, although individuals who survived past 16 years have been recorded [20], its longevity may reach up to 13 years [21] with a median lifespan averaging 5.5 years [22]. Mouse lemurs are strict long-day photoperiodic breeders [23]. At the beginning of the breeding season, females enter oestrus and males compete to priority access to females [24]. Females give birth to 1 to 3 offspring after a 2-month gestation period and nurse infants for approximately 40 days without male parental care. Records of ages at conception for both mothers and fathers and the relative long longevity of captive mouse lemurs give the opportunity to test the “Lansing effect”.

Using a large database on captive mouse lemur’s life history traits, the aim of this study was to examine whether parental age at conception affects the longevity of offspring. We expected a reduced longevity of offspring born from old parents. Finally we tested the potential relationship between parents’ and offspring’s longevity.

## Material and methods

### Animals

To investigate the effects of parental age at conception on offspring lifespan, we analysed the longevity data of mouse lemurs for which the age of both parents at the time of reproduction was known. Data were recorded in the mouse lemur history traits database from a laboratory breeding colony established in Brunoy (UMR 7179 MNHN-CNRS, IBISA Platform, agreement F91.114.1, DDPP Essonne, France) from a stock originally caught near the southern western coast of Madagascar sixty years ago.

Captive conditions were maintained constant with respect to ambient temperature (24–26°C) and hygrometry (55–60%). Animals were fed *ad libitum* on a standardized diet, including fresh fruits, a homemade milky mixture (19.3% proteins, 17.2% lipids and 63.5% carbohydrates) and mealworms. To ensure seasonal reproductive rhythms, animals were routinely exposed to an artificial photoperiodic cycle consisting of 6 months of summer-like photoperiod (LD = 14 h of light/day) followed by 6 months of winter-like photoperiod (SD = 10 h of light/day) [23]. The beginning of the breeding season was induced by the exposure to long days. At the time of LD exposure, groups of 2–3 unrelated males with 1 to 3 females were randomly constituted. Immediately, males entered competition for priority of access to oestrous females, leading to a hierarchy mostly depending on aggressive interactions [25]. During sexual competition, several nest boxes were provided so that animals can escape agonistic interactions from conspecifics and the chase or fight immediately stops when the chased animal enters a nest-box. In the colony, heterosexual groups were restricted to the few days of female estruses. The rules set in the breeding colony for reproductive events included: the limited number of breeding by year, the female age, body mass of sexual partners, maternal lineage and previous reproductive success. Paternity determinations, recorded in the database, allowed calculating the reproductive success of each male within a group. After mating, males were kept in single-sex groups, and females were isolated for gestation, birth and lactation. Litter size and composition were recorded at birth.

### Data analysis

Our analysis was focused on parents (132 dams and 122 known sires) and their offspring (140 males and 138 females) that died naturally.

Paternity determinations have been previously determined by behavioural observations and genetic analyses and were registered in the database. Briefly, DNA samples were collected from ear or skin tissue samples, extracted (with a QIAmp DNA Mini Kit no. 51306—Qiagen) and amplified. Genetic analyses were then conducted using random amplified polymorphic DNA method or microsatellite loci [25–27].

To assess which parameters may influence the longevity of offspring, several parameters for both parents and offspring were selected. Data are presented as mean± SEM.

First, age at conception for both parents was considered. dams’ age at conception averaged 2.7 ± 0.1 years (N = 278), the majority of dams being less than 5 years old; dams aged more than 5 years old represented only 8%. Several dams have had several litters with different sires (N = 19). Sires’ age at conception was significantly higher than that of dams (3.2 ± 0.1 years, df_1/554_, F = 16.0, P < 0.001) and 85% of sires were less than 5 years old. Parents could have offspring from the first breeding season (minimum 260 days), and can reproduce until death like to other prosimians species [18, 19, 28]. In our sample however, due to management reasons, parental age at breeding did not exceed 8 years. In this study, parents were considered young when ≤ 2 years old, adults when > 2 to 5 years old, and aged when > 5 years old. Second, parents’ lifespan was included in the analyses.

For offspring, only young that were alive when 6 month-old were included in this study. Dams’ parity was not included because previous studies demonstrated that this parameter did not influence the size or the composition of the litters, or growth rate during the first month of life [29]. However, size (from 1 to 3 offspring) and type of litter in which offspring were born: male litters (M, MM, MMM), female litters (F, FF FFF) and mixed-sex litters (MF, MMF, MFF) were considered.

Body mass (g) at 30 days, i.e. near weaning time (N = 267), thus providing a clue on maternal investment, as well as at 2 months after birth (N = 273) were included. Age and timing relative to the photoperiodic regimen of natural death were incorporated in the analyses. Lastly, because of the energy costs of mating [30] opportunities for offspring to mate, were also recorded.

### Statistics

Data are presented as means ± SEM. Statistical analyses included Cox proportional hazards model, multi-way analyses of variance using or not a covariate and G tests to test distributions. Multiple pairwise comparisons were made using Tukey’s post hoc test. Relationships between the different factors were tested using linear regression analyses. All statistical analyses were conducted using Systat Software.

### Ethics statement

All the results in this study did not correspond to experimental procedures but are issued from the exploitation of lifespan data collected in the long-term mouse lemur’s captive population maintained for scientific purposes. We adhered to the Guidelines for the Treatment of Animals in Behavioural Research and Teaching [31] and the legal requirements of the country (France) in which the work was conducted. The colony is established under the authorisation of the Direction Départementale de Protection des Populations (DDPP/022-F91-114-1). All procedures to breed mouse lemurs are conducted in accordance with the European Communities Council Directive (86/609/EEC) and are authorized by the Departmental Veterinary Services (Directive 2010/63/UE—Capacity certificate Préfecture de l’Essonne, 04/03/1995). Specifically, for these arboreal primates, housing conditions include cages equipped with branches, various supports, devices to stimulate foraging, and many nesting boxes allowing the animals to express their entire behavioural repertoire. The health and the well-being of captive animals are regularly checked by the animal care keepers and the veterinarian. To improve animal welfare and the enrichment of breeding conditions, meetings of the welfare unit are held every month. When an animal shows signs of poor conditions or signs of social distress, it is isolated and monitored until fully recovered. Lastly, only animals that were found dead were used to conduct longevity research, so effects of anaesthesia, euthanasia or animal sacrifice influenced this study.

## Results

### 1) Offspring longevity and postnatal parameters

Using Cox proportional hazards model, the median lifespan of offspring examined in this study reached 5.5 ± 0.1 years (N = 278) without a significant difference between males (5.53 ± 0.2 years, N = 140) and females (5.46 ± 0.2 years, N = 138, df_1/276_, F = 0.12, P > 0.7). Maximal lifespan reached 12.1 years in males and 11.1 years in females. Natural deaths mainly occurred at the photoperiodic transitions (43%, Gdf_2_ = 13.6, P < 0.010) but were significantly more frequent during long-day photoperiod (df_1/274_, F = 4.96, P = 0.024) independently of sex (df_1/274_, F = 0.01, P > 0.9).

#### Size and type of the litter

Offspring mainly issued from litters of 3 (55%), with litter size of 1 or 2 representing 9% and 36% respectively; the distribution of litter size was identical within sexes (Gdf_2_ = 2.13 P > 0.3). Mixed sex litters were most prevalent (63%) among offspring studied.

Dams’ age at conception had an impact on the size of the litter produced (r = 0.159, P = 0.008), with young dams producing less numerous triplets than older dams (45% versus 66% respectively—df_2/275_, F = 3.25.18, P = 0.039). The type of the litter was independent of the dams’ age (df_2/275_, F = 0.002, P > 0.9). By contrast, whereas sires’ age had no impact on litter size (r = 0.092, P > 0.1), younger sires produced less numerous male-biased litters (df_2/275_, F = 4.3, P = 0.015).

Accordingly, the size and the type of the litter were dependent on both parents with adult pairs producing more numerous offspring (63%) in more numerous male-biased triplets (litter size df_2/275_, F = 4.09, P = 0.022 and litter type df_2/275_, F = 5.99, P = 0.003).

**Fig 1:**
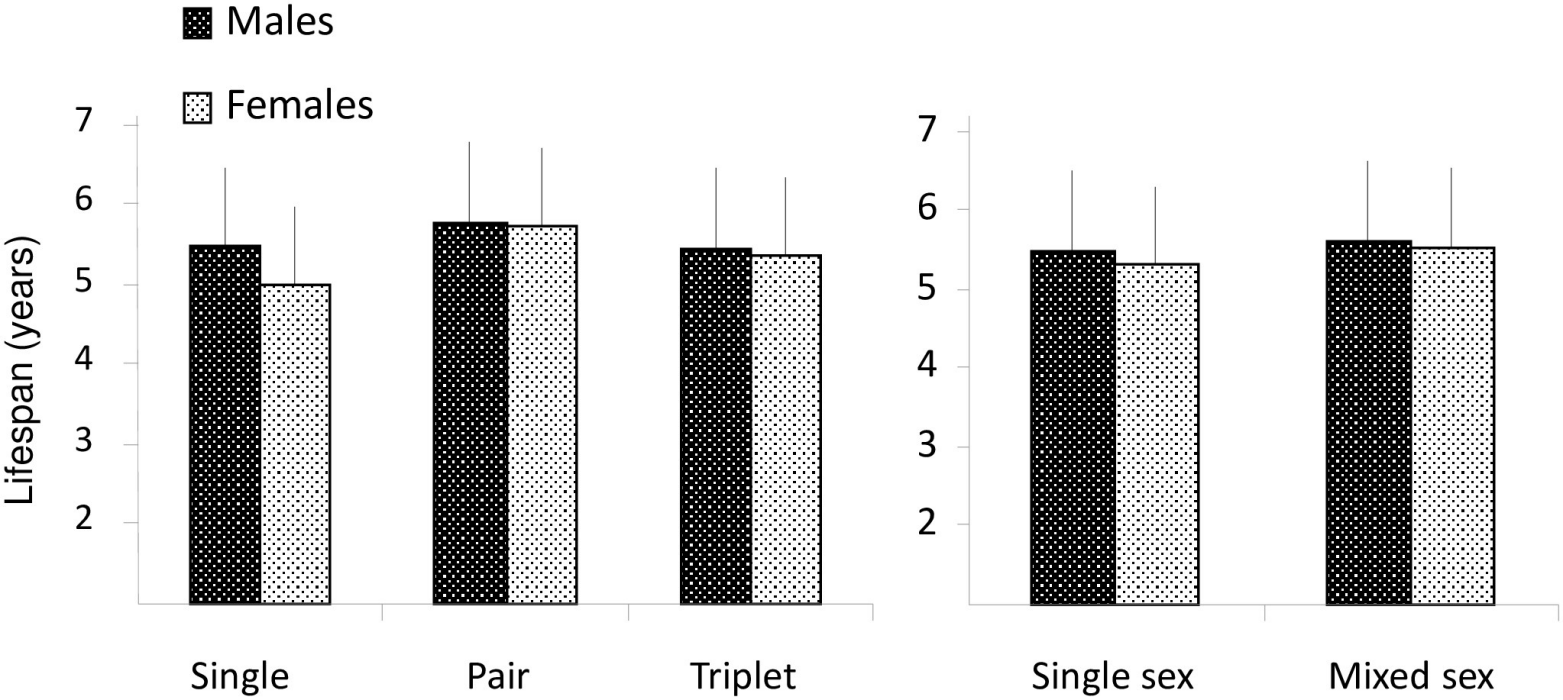
Lifespan (means ± SEM in years) in female and male offspring according to the size (left panel) and the type (right panel) of the litters.

But offspring longevity was independent of the size (df_2/275_, F = 0.73, P = 0.5) and of the type of the litter (*df*_2/274_, F = 0.08, P > 0.7), whatever the offspring sex (*df*_2/274_, F = 0.08, P > 0.9—Fig 1).

#### Post natal body mass

Offspring body mass reached prior to weaning time (30 days) averaged 32.1 ± 0.4g (N = 266—Table 1). It was independent of both dams ‘age (r = 0.043, P > 0.7) and sires’ age (r = 0.020, P > 0.8), but it was strictly linked to the size of the litter. Indeed, body mass of animals born in a triplet was significantly lower (*df*_2/260_, F = 14.1, P < 0.001) than that of animal born alone or in pairs, independently of sex (*df*_1/260_, F = 0.09, P > 0.8) and of litter type (*df*_2/263_, F = 1.3, P > 0.2).

**Table 1:** Body mass (g, mean ± E) of female and male offspring at weaning time (30 days) and 60 days after birth depending on litter size.

	Body mass (30 days)		Body mass (60 days)	
Litter size	Females	Males	Females	Males
Single	31.9 ± 2.8 (9)	34.9 ± 2.2 (13)	50.8 ± 2.4 (9)	58.6 ± 2.2 (13)
Pair	35.1 ± 0.8 (51)	33.5 ± 0.9 (47)	53.1 ± 1.2 (51)	52.3 ± 1.2 (49)
Triplet	30.4 ± 0.8 (75)*	30.3 ± 0.7 (71)*	49.3 ± 1.0 (77)*	47.9 ± 0.9 (74)*
Mean	32.3 ± 0.6 (135)	31.9 ± 0.6 (131)	50.8 ± 0.8 (137)	50.5 ± 0.7 (136)

* Significant differences between triplets and other litters (P < 0.001).

Offspring body mass averaged 50.6 ± 0.5 (N = 273 Table 1) 2 months after birth and did not differ between sexes (P > 0.92). It remained however dependent on the size of the litter (*df*_2/267_, F = 11.6, P < 0.001) but not of litter type (P > 0.1) although male offspring born without littermates were significantly heavier (df_2/270_, F = 12.3, P < 0.001). Lastly, maternal investment in terms of offspring’s body mass did not depend on dams’ age at conception (respectively P > 0.7 at 30 days, P > 0.8 at 60 days).

Offspring longevity was not linked to their body mass at weaning for both males (r = 0.109, P > 0.2 N = 131) and females (r = 0.111, P = 0.2, N = 135). Likewise the body mass reached by offspring 2 months after birth was unrelated to their lifespan whatever the sex (r = 0.057, P > 0.3, N = 273).

### 2) Offspring’s longevity and reproductive investment

Among female offspring, no difference in longevity was observed between females that had access to reproduction at least once and those that did not come in contact with males (respectively 5.12 ± 0.2 years, N = 55 versus 5.62 ± 0.2 years, N = 83—df_1/136_, F = 2.71, P = 0.1). By contrast, males having at least one opportunity to mate had significantly longer longevity than the others (respectively 6.04 ± 0.2 years N = 30 versus 4.26 ± 0.3 years, N = 102, df_1/138_, F = 17.6, P < 0.001).

### 3) Offspring ‘s longevity and parental age at conception

**Fig 2:**
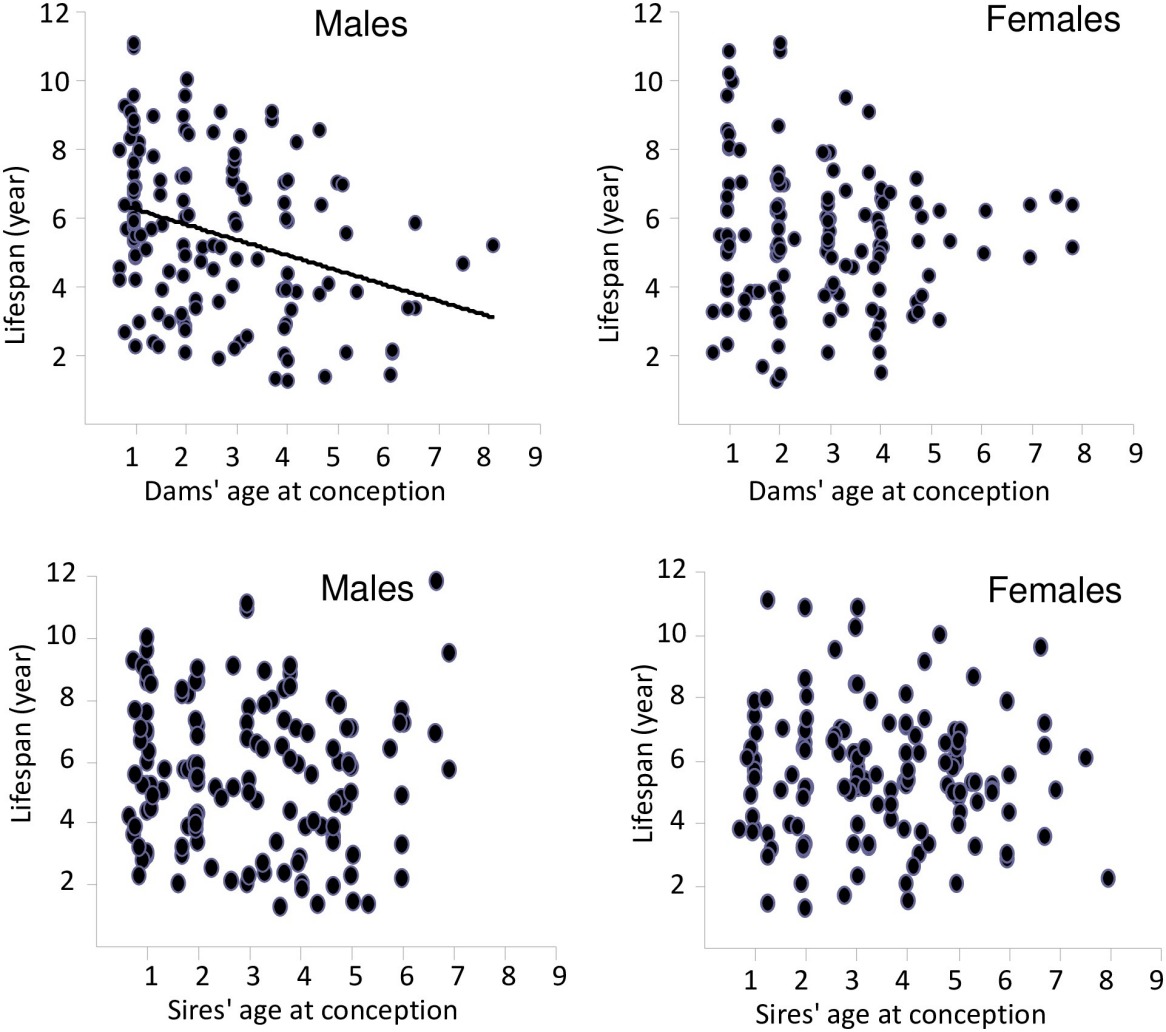
Relationship between offspring longevity in males (left panel) and females (right panel) according to the age at conception of dams and sires. Only, a significant correlation was observed between age at conception of dams and longevity in male offspring (r = —0.309, P < 0.001).

Dams’ age at conception was negatively correlated to offspring’longevity for males (r = —0.309, P < 0.001) but not for females (r = 0.104, P = 0.226- Fig 2). However, Regardless of their sex (P > 0.7), offspring born from 1 year-old dams (primiparous) have a significantly longer longevity than those born from multiparous dams whatever their age (respectively 6.40 ± 0.3 years, N = 67 versus 5.23 ± 0.1 years, N = 211, df_1/274_, F = 14.8, P < 0.001).

By contrast, the sires’ age at conception did not influence offspring’ longevity for both males (r = 0.057, P > 0.5) and females (r = 0.027, P > 0.8—Fig 2).

**Fig 3:**
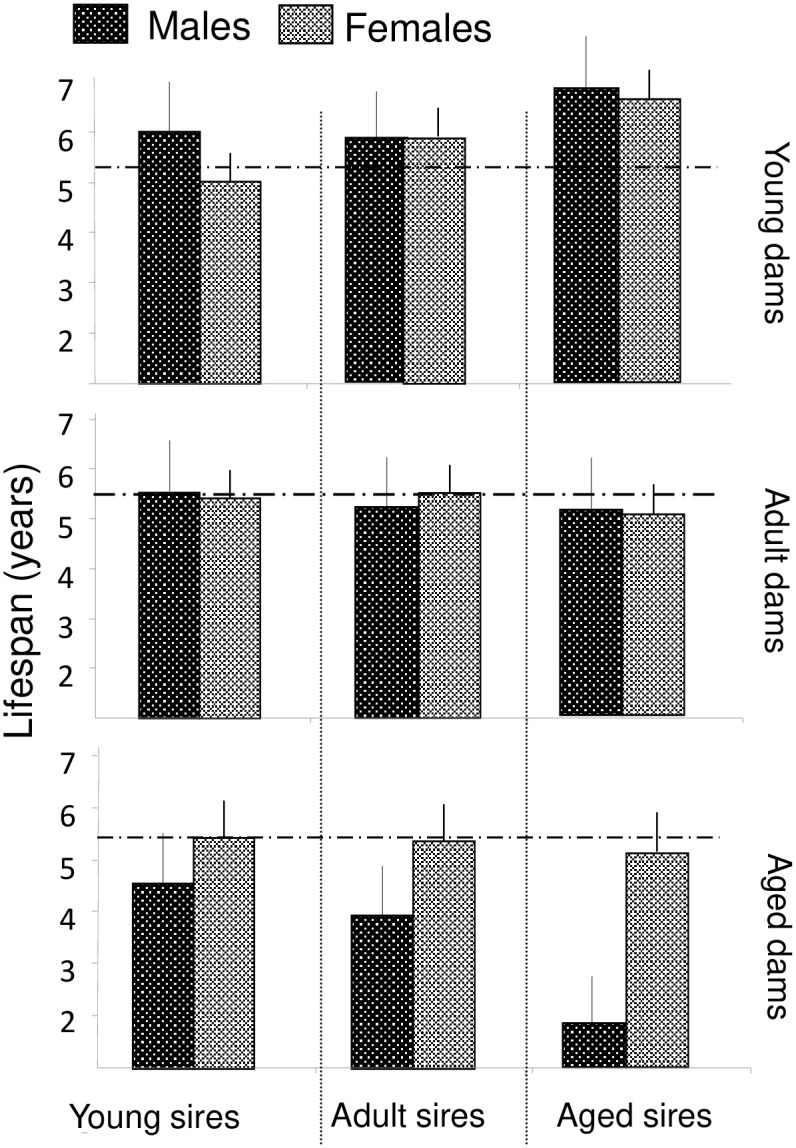
Lifespan (means ± SEM) of male and female offspring according to the parents’ ages within a breeding pair. The broken line indicates the mean survival (5.5 years).

Since sires’ age at conception did not influence the lifespan of offspring, the effect of the parents within a pair on offspring’s longevity would be mainly dependent of the dams’ age. To assess the differential effect of the parents’ age within a pair, we used Cox proportional hazards model with age at conception of both parents as covariate factors. The male offspring longevity was negatively affected by the dams’ age at conception (P < 0.001) whatever the sires’ age (P > 0.9). The female offspring longevity was independent of sires’ age (P > 0.5) and of dams’ age (P = 0.4). Whatever the classes of parents’ age (Fig 3), only dams ‘ age at conception influenced the longevity of offspring (males df_1/136_, F = 13.6, P < 0.001) but not of female offspring (df_1/134_, F = 1.4, P = 0.5). The minimum longevity of male offspring was observed when both parents were old (P < 0.02). In addition, when analysing the difference in parental age at conception, the longevity of male offspring was significantly shorter when dams were older than sires (r = 0.191, N = 140, P = 0.024). No such correlations were observed for female offspring (dam r = 0.060, P > 0.4, N = 138).

Lastly, when considering the potential relationship between parental longevity and that of their offspring, a significant difference appeared according to sex. The lifespan of male offspring was unrelated to the longevity of their respective mother (r = 0.006, P > 0.9) and sire (r = 0.078, P > 0.3). By contrast, the longevity of female offspring correlated significantly with both their mother (r = 0.168, P = 0.048) and their sire (r = 0.213, P = 0.012).

## Discussion

The longevity of captive mouse lemurs may reach up to 13 years with a 50% survival time around 5.5 years [21, 22] and with no significant difference between sexes. Mouse lemurs are highly seasonal breeders and can reproduce throughout all their life even at an advanced age [19, 28]. Results demonstrated that mothers ‘age at conception affected only the longevity of male offspring while the fathers’ age at conception had no influence on longevity of offspring, whatever their sexes. Like to ruffed lemurs [18], the absence of an effect of fathers’ age suggests that male reproductive senescence is absent in male mouse lemurs. As the mother aged, the survival of male offspring decreased with a minimum when both parents were old. More, as previously described in human [12, 32, 33], only the longevity of female offspring was significantly related to the longevity of both parents.

Among vertebrate species, the effect of mother’s age on pre-adult survival has been well documented [1, 2]. With ageing, a decrease in fertility and in maternal investment is considered to be the key for reduced survival of offspring. Under captive conditions with reduced environmental stress, age does not affect either fertility or maternal investment in female mouse lemurs. As mother age, litter size increases and postnatal growth of offspring remains unaffected. Thus, ofspring survival in adulhood is not a result of an inadequate maternal investment or on postnatal conditions in captive mouse lemurs.

In our sample, the mother’s age at conception was negatively correlated with male offspring longevity, but the father’s age at conception had no impact on either sex. This strongly suggests a predominant role of genetic load from the mother.

The effect of parental age on offspring longevity is generally attributed to a direct age-related deterioration of the germ cells: DNA mutation, DNA methylation, shortening of telomeres [34]. Within age-related deteriorations, the telomere length (TL) is recognized as the most suitable biomarker of aging [3] and telomere inheritance appears to be paternal in mammals [4]. A large comparative study on mammalian species, including humans, showed clear relationships between age-related shortening of TL and lifespan [4, 35]. However, the age-related TL shortenning appears to be sex- and species-dependent [36–39]. If strong evidence exists for genetic inheritance of TL, conclusions on relationship between parental age at conception and TL of offspring depend on the species studied. In several studies (mostly birds), TL shortens as parents age and predicts offspring TL with sex-specific differences in TL inheritance [6, 8, 40–47]. By constrast, a weak or no relationship between TL and parental TL has been observed in other species [45–48].

Telomere biology in mouse lemurs seems an exception among primates. The average telomere length is among the longest reported for primate species and, no detectable TL shortening with ageing was detectable [49]. Like to hamsters during short day period [50], mouse lemurs use daily torpor, which may contribute to an increase in TL. Moreover, in small-bodied species, the seasonal variation in sperm production might explain the lack of an effect of parental age on TL [46]. In male mouse lemurs, a greater sperm production, the presence of telomerase activity in testes and social dominance required for successful mating may all contribute to maintenance of TL [51, 52]. Dominant males (i.e. fathers) would invest more heavily in soma reparation and TL maintenance as suggested by the longer lifespan of males that were offered the opportunity to reproduce successfully at least once. All these characteristics could explain the lack of effect of fathers’ age at conception on offspring longevity. Similarly, for female mouse lemurs, the use of torpor, the high reproductive seasonality and the protective role of oestrogens could contribute to TL maintenance over life.

Why mothers’ age at conception affects only male offspring remains however a question. As supported by Entringer et al. *2015* [53], oestrogens levels of the mother would predict TL of offspring. With ageing, levels of oestrogens in female mouse lemurs significantly decrease and male-biased litters are more frequent [54]. But, this decrease does not impact females’ fecundity or female offspring longevity. In humans mitochondrial DNA content is higher in females and no deterioration was observed at advanced maternal age [55]. Thus, offspring from aged mothers would not suffer from lesser portion of maternal genetic load. However, in our study, the lifespan of male offspring from aged mothers have a reduced lifespan compared to those born from younger mothers, whatever the age of the fathers. This sex-specific effect of aged mothers on male offspring longevity could rely to others parameters such as the composition of the litters [56], age-related maternal behaviours or social interactions later in the weaning period. As a consequence, these results could allow better management of the captive colony by removing old females from the breeding pairs and by selecting young females from long-lived lineages.

According to several studies [12, 57, 58], life-history trajectory of offspring could vary according to mothers’ age suggesting potential trans-generational effects of maternal age. For female mouse lemurs, it has been already described that, in the highly favourable conditions of captivity, there was no cost of reproduction for longevity [59]. In several species, offspring from older mothers show changes in their lifetime reproductive success, associated or not with a shorter lifespan [57, 60, 61]. Further work is needed to know whether reproductive success in female mouse lemurs is affected by maternal age at conception. It would be of interest to follow the longevity of all female offspring from a maternal line to decipher the inheritance of female longevity.
